# Neoplasia-Associated Wasting Diseases with Economic Relevance in the Sheep Industry

**DOI:** 10.3390/ani11020381

**Published:** 2021-02-03

**Authors:** Marcelo De las Heras, Marta Borobia, Aurora Ortín

**Affiliations:** Departamento de Patología Animal, Facultad de Veterinaria, Instituto Agroalimentario de Aragón-IA2, Universidad de Zaragoza-CITA, C/Miguel Servet 177, 50013 Zaragoza, Spain; mborobia@unizar.es (M.B.); aortin@unizar.es (A.O.)

**Keywords:** small intestinal adenocarcinoma, ovine pulmonary adenocarcinoma, jaagsiekte sheep retrovirus, enzootic nasal adenocarcinoma, enzootic nasal tumour virus

## Abstract

**Simple Summary:**

Neoplasia is a common cause of weight loss and emaciation. Despite its relative infrequency in sheep, there are three neoplastic diseases with special relevance. Small intestinal adenocarcinoma (SIA), ovine pulmonary adenocarcinoma (OPA) and enzootic nasal adenocarcinoma (ENA) are three neoplastic diseases with economic impact in the sheep industry. They mostly occur at ages under common production cycles, have epidemiological relevance in sheep rearing countries, and two of them (OPA and ENA) have an infectious aetiology. SIA occurs elsewhere in the world but has a special economic impact in Australia and New Zealand. OPA and ENA have relevant economic significance in most continents but have not been recorded in Australia and New Zealand. In this review, we focus on the epidemiology, clinicopathological features, pathogenesis and the diagnostic tools currently available for the diagnosis of these three neoplastic diseases.

**Abstract:**

We review three neoplastic wasting diseases affecting sheep generally recorded under common production cycles and with epidemiological and economic relevance in sheep-rearing countries: small intestinal adenocarcinoma (SIA), ovine pulmonary adenocarcinoma (OPA) and enzootic nasal adenocarcinoma (ENA). SIA is prevalent in Australia and New Zealand but present elsewhere in the world. This neoplasia is a tubular or signet-ring adenocarcinoma mainly located in the middle or distal term of the small intestine. Predisposing factors and aetiology are not known, but genetic factors or environmental carcinogens may be involved. OPA is a contagious lung cancer caused by jaagsiekte sheep retrovirus (JSRV) and has been reported in most sheep-rearing countries, resulting in significant economic losses. The disease is clinically characterized by a chronic respiratory process as a consequence of the development of lung adenocarcinoma. Diagnosis is based on the detection of JSRV in the tumour lesion by immunohistochemistry and PCR. In vivo diagnosis may be difficult, mainly in preclinical cases. ENA is a neoplasia of glands of the nasal mucosa and is associated with enzootic nasal tumour virus 1 (ENTV-1), which is similar to JSRV. ENA enzootically occurs in many countries of the world with the exception of Australia and New Zealand. The pathology associated with this neoplasia corresponds with a space occupying lesion histologically characterized as a low-grade adenocarcinoma. The combination of PCR and immunohistochemistry for diagnosis is advised.

## 1. Introduction

The relative infrequency of the occurrence of neoplasms in sheep seems to be apparent specially when compared with infectious or parasitic diseases. However, an unbiased prevalence and distribution data of tumours in sheep is difficult to obtain due to differences in sheep breeds, geographical location, survey parameters and management systems all around the world [1,2]. There is a paucity of published surveys, and they are mostly retrospective abattoir-based, academic or from diagnostic centres. In addition, the onset of neoplasms increases with age, and sheep under common production cycles are culled before the natural lifespan has elapsed [2]. Despite this, some types of ovine tumours occur frequently in some countries where the sheep industry is important and appear during the commercial lifespan of sheep.

Most neoplastic diseases cause weight loss and emaciation in the affected animal. However, there are neoplastic wasting diseases affecting sheep with some particular aspects, which give them a special consideration. We selected for this review three neoplastic diseases: small intestinal adenocarcinoma (SIA), ovine pulmonary adenocarcinoma (OPA) and enzootic nasal adenocarcinoma (ENA). These three diseases are mostly recorded at ages under common sheep production cycles, up to 5–7 years. OPA and ENA are most often detected in sheep that are 2–4 years of age, and SIA is mostly detected in animals aged 5–7 years. The second characteristic is their epidemiological relevance with economic impact in the sheep industry. Thus, OPA is endemic in countries such as Peru, Scotland, South Africa and Spain, and SIA is highly prevalent in New Zealand and Australia [2]. The third element is the causative relationship with betaretroviruses for two of them, OPA and ENA, which categorizes them as infectious diseases [3]. An additional interest of these diseases is their similarities to respiratory or digestive human tumours, which make them useful models for comparative oncology [4,5].

In this review, we focus on the epidemiology, clinicopathological features, pathogenesis and the diagnostic tools currently available for the diagnosis of these three neoplastic diseases.

## 2. Small Intestinal Adenocarcinoma

Adenocarcinoma of the small intestine (small intestinal adenocarcinoma, SIA) is a naturally occurring neoplasia of the small intestine of sheep which is highly prevalent in some of the most important sheep-rearing countries.

### 2.1. Epidemiology

SIA is reported with high prevalence in some countries, such as New Zealand and Australia, but is also commonly recorded in Scotland, Norway and Iceland; meanwhile, sporadic cases have been reported from elsewhere in the world [2,6,7,8,9]. Although with variations depending on the geographic area, prevalent figures obtained from abattoir studies ranged from 0.2 to 1.6% in inspected adult sheep in New Zealand, Scotland and Australia [6,7]. Therefore, this prevalence justifies the economic relevance of SIA for leading sheep-rearing countries. Studies carried out in New Zealand and Australia described SIA in Romney, Merino, British breeds and crosses with Merino [6,10,11,12], with a significantly higher prevalence in British breeds [6]. However, SIA has been described in other breeds elsewhere in the world, and not enough data are available supporting the fact that breed is a risk factor for this neoplasia. SIA occurs at all ages but is mostly recorded in animals 5–7 years old [10,11,12,13], under the age of common sheep production cycles.

### 2.2. Clinical Features and Pathology

In most studies, recorded cases of SIA did not include clinical descriptions of the affected sheep because they were dead when examined or the investigations were abattoir based. In studies where clinical descriptions were detailed, first signs were not specific, and sheep looked dull, separated from the mob during delivery, walked with a rolling gait, showed signs of constipation and eventually died [12]. Some animals developed abdominal pain, distension, ascites [9,10,12,13] and paracentesis demonstrated by large amounts of clear fluid [12]. In many cases, sheep bearing SIA are not clinically detected and animals only show a graduate loss of appetite and weight during a variable period ranging from a few weeks up to six months [9,12,13].

**Figure 1:**
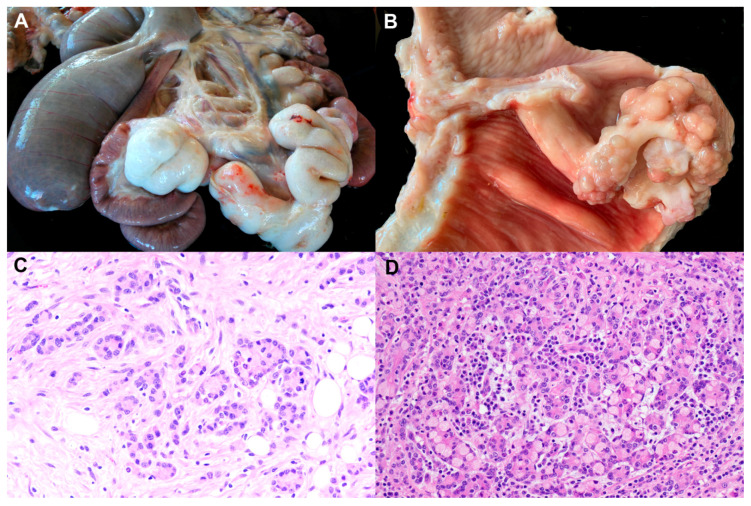
Gross pathology and histopathology of the small intestinal adenocarcinoma. (**A**) Sheep 5 years old. Serous surface on distal third segment of the small intestine. Firm and white mass extending over serosa of convoluted intestinal segment. Plaited whitish cords are extended on the mesentery. (**B**) Sheep 5 years old. Section of the intestine showing single polypoid pedunculated mass causing stricture. Lumen of the intestine greatly dilated is observed proximal to the mass. (**C**) Histopathology of an intestinal sample from A. Acinar and tubular structures of various sizes lined by cuboidal or columnar epithelial cells replace the intestinal mucosa. Small groups or cords of epithelial cells are also seen. Moderate desmoplastic reaction together with lymphocyte infiltration is observed. Hematoxylin-eosin. 200×. (**D**) Histopathology of an intestinal sample from B. Signet ring cells characterized by mucin-filled cytoplasm; peripheralized crescent-shaped nuclei are numerous in this area of the tumour. Hematoxylin-eosin. 100×.

At post-mortem examination, clinically affected sheep showed emaciation together with variable degrees of ascites: from 200 mL to several litres of yellowish fluid [9,10,13]. The edema fluid can be extended to the thoracic cavity [13]. One streaking feature observed at necropsy was the location of the tumour. The primary site of the tumour was in most cases in the middle third of the small intestine (jejunum), followed by the distal third and more rarely in the duodenum [4,9,10,11,13]. The tumour was identified by the presence, at those sites, of a dense, firm and white mass extending over serous surfaces of the intestine along the mesentery [4,9,10,11,13] (Figure 1A).

However, in many cases, dissemination beyond these sites has occurred, reaching other organs such as the stomach or liver, and it is identified by white spots, plaques or diffuse sheets of dense tissue rising above the affected areas [4,10,11,13]. Occasionally, similar picture can be observed over the diaphragmatic pleura and pericardium [13]. The mesenteric lymph node chain was generally enlarged and much firmer than normal [10,11,13]. Lymph node metastases were detected in the majority of tumours [4]. In advanced cases, when sections were obtained from affected areas of the intestine, the wall showed a thick firm white layer replacing serous coat, and the lumen was filled either with yellowish gelatinous substance blood stained or large clots of blood causing complete obstruction [10,11]. Longitudinal sections of the intestine of early and advanced cases showed single or multiple polypoid masses with thick peduncle projecting into the lumen causing variable degree of obstruction [4,10,11,13]. Proximal to the stricture, the lumen of the bowel was greatly dilated and filled with digesta [4,9,10,11,13], and distal to this point, the small and large intestine contained little or no food material [11] (Figure 1B).

The histopathology of the SIA has been studied by several authors, and descriptions are very similar among different countries [4,7,9,10,13]. Intestinal architecture is remarkably altered and tumour cells varying in size are present through all layers of the intestinal wall and follow an acinar pattern or are in the form of closely packed cells [4,10]. Neoplastic cells are large, moderately pleomorphic, polyhedral cells with well-defined borders. Cells contain a large, round and central nucleus with prominent nucleoli. Cell cytoplasm includes variably sized vacuoles that often contain mucus [4,7]. There is coexistence between more differentiated types of epithelial cells, such as enterocytes and goblet cells organized in acini, or in tubular fashion with less differentiated types in closely packed cells [8,9,10]. Generally, SIA is histologically graded as moderate or poorly differentiated [4]. In addition, groups of signet-ring cells are also seen, but they are not a prominent feature in most descriptions [4,9,10,13]. Mitotic figures are rare [8] and have been estimated in 2–5 mitosis per high power field. Necrosis is a common event [4] (Figure 1C,D).

The latest histologic classification of SIA in WHO histologic classification of tumours of the lower alimentary tract indicates that signet-ring cell carcinoma is the most frequent pattern [14]. Signet-ring cell carcinoma is defined in the same publication as a malignant epithelial tumour which is composed in more than a half of these cells. However, although signet-ring cells can be part of the cells composing the SIA, several authors indicate that these cells are present in all tumours, but they are not a predominant feature of the SIA in sheep, and signet-ring cells are very few in sheep tumours [4,8,9,13]. Some authors have indicated that SIA of sheep have features of several types of histological patterns listed on the WHO classification of 1976 [15], and the most appropriate description for SIA is that of tubular adenocarcinoma [8,9].

### 2.3. Risk Factors

The predisposing factors or aetiological relationship of the SIA are not known. The high prevalence found in some geographic areas, such as New Zealand, indicates a link with environmental carcinogens or genetic predisposition. Thus, in some epidemiological studies, the tumour was more prevalent in British breeds than in fine wool sheep, or in areas with increased density [6,16]. This genetic predisposition has also been suggested in reports of cases of closely related sheep in one flock [17]. In other studies, exposure to herbicides (phenoxy and picolinic acid) has been associated with a significant increase in the rate of SIA in some breeds [16]. Despite these studies, no conclusive evidence of the relationship of SIA with either genetic factors or environmental carcinogens has been found.

SIA has not been found to be associated with frequent infections, such as herpesviruses, *Helicobacter* spp. or *Mycobacterium avium* subsp. *paratuberculosis* [18], and screening for virus production in explant cultures from sheep SIA cells proved negative [19]. In addition, the presence of *Bacteroides fragilis* toxin gene, a molecular marker of colonic carriage of enterotoxigenic *Bacteroides fragilis* (ETBF) in humans, which is related to colon cancer, was investigated in SIA. There was no apparent association between the carriage of ETBF and the presence of SIA [20].

SIA has been proposed as a model of human intestinal cancer [4], and some studies have indicated an altered expression of important proteins also dysregulated in human colon cancer [21]. However, studies in SIA on the expression of mismatch repair proteins do not support that germline defects in their corresponding genes are predisposing factors, as happens in human colon cancer [22].

### 2.4. Diagnosis

There are no clinical diagnostic tests for SIA, and the confirmation requires histopathological analysis of the tumour samples.

## 3. Ovine Pulmonary Adenocarcinoma

Ovine pulmonary adenocarcinoma (OPA, ovine pulmonary carcinoma, sheep pulmonary adenomatosis and jaaagsiekte) is a contagious lung cancer of sheep caused by jaagsiekte sheep retrovirus (JSRV) [23,24]. The disease, which is invariably fatal, is a wasting disease clinically characterized by an afebrile progressive respiratory condition as a consequence of the development of lung adenocarcinoma, since JSRV induces the neoplastic transformation of secretory epithelial cells of terminal bronchioles and alveoli [25,26]. OPA has been reported in many of the sheep-rearing areas of the globe and causes significant economic losses, as the implementation of effective strategies for the control and eradication is not an easy task due to the lack of vaccines and the difficulty in identifying preclinical stages and, specially, lesion-free infected animals [27,28]. No vaccines for OPA have been developed yet. An unusual feature of JSRV infection in sheep is the absence of detectable antibody or T cell responses following natural or experimental infection. However, antibodies specific for JSRV capsid protein were induced by inoculation of recombinant proteins in adjuvants. Studies are underway to characterize and improve these responses and to determine whether they are protective against infection with JSRV and/or the development of OPA [27].

### 3.1. Epidemiology of OPA and JSRV Infection

The first descriptions of OPA were made more than 100 years ago in South Africa and Britain and, since then, the disease has been reported in many countries elsewhere in the world with the exception of Australia and New Zealand. The disease was eradicated in Iceland in the 1950s by drastic slaughter measures [26].

Under natural conditions, clinically apparent OPA mostly appears in animals 1–4 years old, although the disease can occur at all ages, and there is no clear evidence of sex or breed susceptibility [25]. The incubation period for the development of clinical disease following natural infection may last from months to years, but is shorter (6–8 months) in flocks where OPA is not endemic. On the other hand, when very young lambs are experimentally infected, clinical signs tend to appear in 3–6 weeks or even before [25,26].

The mortality rate in OPA affected flocks varies depending on how long the infection has been present. In the first years after introduction, the infection mortality rates can reach 30–50% (as during the OPA epidemics in Iceland in the 1930s), but rates drop to 1–5% when the disease becomes endemic [25,27]. The prevalence of OPA appears to vary between countries and is endemic in some of them, such as Peru, Scotland, South Africa and Spain [26]. A study conducted in a Spanish abattoir recorded visible OPA lesions in 0.3–1.4% of sheep slaughtered in a year [29], and high figures were also obtained in an abattoir study in Edinburgh [30], indicating that OPA prevalence may be underestimated. Results from a longitudinal survey in two OPA endemic flocks carried out in Scotland are in accordance with this. Around 30% of the sheep had histologically confirmed OPA lesions, but the annual losses attributable to OPA varied between 2 and 10% [26]. These findings were obtained by clinical observation and histopathological analysis and, therefore, do not reflect the prevalence of JSRV infection in OPA-affected flocks. The lack of a detectable specific immune response against JSRV in infected animals [31,32] prevented the development of serological tests, and the acquisition of certain knowledge about this issue was not possible till the arrival of molecular techniques for the specific detection of JSRV, which demonstrated the tissue distribution of the virus outside the OPA lesion [33]. In this way, JSRV proviral DNA could be detected by PCR in lymphoid tissues and peripheral blood mononuclear cells (PBMC) in clinically OPA affected sheep, in animals with preclinical OPA lesions and in lesion-free infected animals [34,35,36,37,38]. The PCR test for the detection of JSRV proviral DNA in blood cells has been used in epidemiological studies, pointing to an incidence of JSRV infection in OPA-affected flocks much higher than previously believed [36,38]. In a study in which a Spanish commercial flock was tested periodically over three years, at the end of the study, the virus had been detected in 50% of the animals [38]. JSRV could be detected in animals of all age ranges, but the highest incidence (80%) was found in animals that were under one year old when the study began [38]. Despite this, only a minority of animals that tested positive developed OPA lesions (17%), many of which were subclinical, since 40% of animals with OPA lesions at necropsy were not clinically affected [38]. This is in accordance with a previous study indicating that many OPA cases can remain subclinical at the end of the sheep’s commercial lifespan, and induction of OPA is not a common outcome of naturally occurring JSRV infection [36].

It is generally accepted that the respiratory route is the most important natural mode of transmission for JSRV [25]. In recent years, the contribution of colostrum and milk (C/M) to the spread of infection in commercial sheep farms has also been investigated. Epidemiological studies demonstrated that JSRV infection can occur perinatally or in the first few months of life in lambs [36,38], and the prevalence of JSRV infection in this age range can be particularly high in commercial flocks, with 30% of lambs destined for replacement found to be JSRV blood positive in one single PCR test [38]. In addition, a survey carried out in a flock with a high prevalence of OPA showed that an important reduction in the incidence of the disease was possible by creating a new flock with lambs separated at birth from their mothers and reared artificially, suggesting that C/M could be relevant for the transmission of JSRV under natural conditions [39]. Further studies demonstrated that colostrum and milk can transmit JSRV to lambs. The presence of JSRV proviral DNA was demonstrated in somatic cells from colostrum in sheep belonging to OPA-affected flocks, and the JSRV provirus was detected in the blood of lambs artificially fed with infected C/M [40]. Evidence of the relevance of this route of JSRV transmission to lambs under natural conditions has been provided by the detection of JSRV in Peyer’s patches and/or mesenteric lymph nodes in 25% of the lambs (between 12 h and 10 days of live) naturally fed by blood infected but asymptomatic ewes [41]. To date, no other details of this way of transmission for JSRV are known. Based on current data, this route of transmission should be taken into account in the design of control strategies, although the possibility that C/M transmission of JSRV to lambs can result in OPA development has not been explored yet.

### 3.2. Clinical Features and Pathology

**Figure 2:**
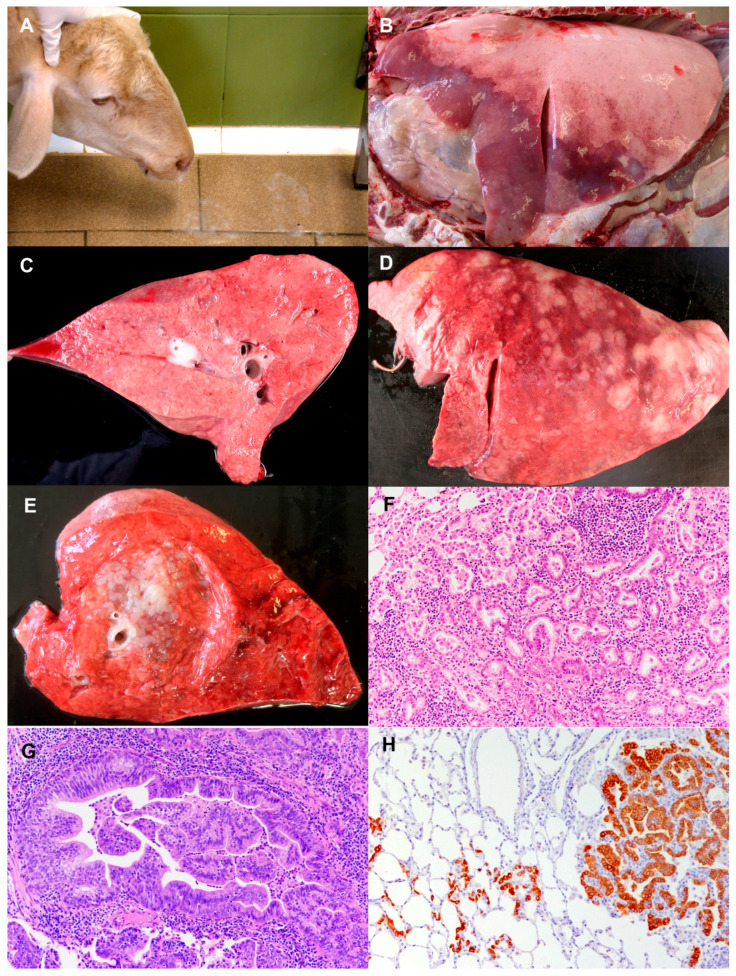
Ovine pulmonary adenocarcinoma (OPA): clinical signs and pathology. (**A**) Four year old sheep showing OPA clinical signs. When head is lowered, frothy sero-mucous fluid comes from the nostrils and pours over the floor. (**B**) Post-mortem examination of thoracic cavity. Classical OPA form. Left lung with cranio-ventral areas purple in colour and consolidated. The rest of the lung shows an increase in volume and is lighter in colour. (**C**) Right caudal lung section. Classical OPA. Lung section showing granular appearance foci and foamy fluid pouring from main airways. (**D**) Left lung from a sheep showing atypical OPA form. Multiple white nodules of various sizes distributed throughout the lung surface. (**E**) Section of lung from right caudal lobe. Atypical OPA. Multiple white nodules with various sizes: bigger ones coalescing close to a central main bronchus and others expanding to close lung areas. (**F**) Histopathology of OPA lesion. Alveolar epithelial cells proliferate following a lepidic pattern. Alveolar wall is replaced by cuboidal cells into a tubular or papiliform pattern. Tumour stroma is infiltrated mainly by lymphocytes. Numerous macrophages are filling alveolar lumens in the tumours and in the close areas. Hematoxylin-eosin. 100×. (**G**) Histopathology of OPA lesion. Bronchiole with epithelium transformed into papiliform growth almost completely filling the lumen. Hematoxylin-Eosin. 100×. (**H**) Immunohistochemistry using a mouse monoclonal antijaagsiekte sheep retrovirus envelope (JSRV Env) protein. Cellular membranes and cytoplasms of neoplastic cells of a tumour nodule are labelled. In the adjacent alveoli, positive cells are observed either isolated or in small groups. 100×.

OPA clinically affected sheep show signs of progressive afebrile respiratory disease associated with cachexia caused by the growth of lung adenocarcinoma. When the lung tumour is very small, the disease is subclinical, but as the tumour becomes extensive enough to interfere with lung function, dyspnoea and moist respiratory sounds caused by the accumulation of fluid in the respiratory airways are detected [25]. In the final stages of the disease, variable amounts of frothy sero-mucous fluid (from 10–40 mL to as much as 400 mL) [25,42] are discharged from the nostrils when the hindquarters are raised (“wheelbarrow” test) or the head is lowered (Figure 2A), which is considered an OPA characteristic sign [43]. Affected sheep remain alert, afebrile and have a good appetite, but progressive loss of weight is evident, and death inevitably occurs within a few weeks of the start of the clinical disease as a result of compromised respiratory function caused by tumour enlargement. However, the clinical course can be shortened, and fever appears if bacterial infections become superimposed. The concurrence of other diseases can also affect the clinical outcome of the disease. Maedi-visna has been frequently reported, and it results in a worsening of the clinical signs and a precipitation of the disease course [25].

Two pathological forms of OPA have been described in the literature, classical and atypical [25]. In the classical presentation, at necropsy, the lungs do no collapse when the chest is opened and are enlarged. The neoplastic lesions can occur in any part of the lungs, but cranio-ventral parts are more frequently involved. They are grey or purple in colour, do no protrude significantly on the surface and have an increased consistency (Figure 2B). The cut surface of the tumour lesion has a granular appearance and is moist, and a frothy fluid pours from the bronchioles and bronchi with slight pressure (Figure 2C). Relatively often, this tumour lesion pattern may be overshadowed by concurrent lesions of bacterial pneumonia, abscesses or maedi. In contrast with the classical form, the atypical presentation tends to be more nodular in both early and advanced tumours. The nodules may be solitary or multiple and mainly located in the diaphragmatic lobes. They are pearly white in colour and have a very hard consistency (Figure 2D). A section of the tumour lesions shows that they are very well demarcated from the surrounding parenchyma, and their surface looks dry (Figure 2E). The tracheobronchial and mediastinal lymph nodes may not show any visible changes or may be slightly or clearly enlarged, and may occasionally present small metastases [25]. More rarely, metastases in distal organs, such as the liver, kidney, heart, skeletal muscle, digestive tract, spleen, skin and adrenal glands, have been observed [25,27,44]. Both forms, classical and atypical, may be present in a flock and in individual sheep, and intermediate and mixed forms have been described. Classical and atypical forms may represent two extremes of the disease spectrum, rather than two separate forms. Descriptions of experimentally induced OPA are compatible with the classical forms observed in natural conditions, but atypical forms have not been reported in experimental conditions [25].

Histological examination of OPA natural cases reveals the presence of neoplastic proliferation foci of epithelial cells in alveolar and bronchiolar areas. These proliferations have a papillary and acinar appearance and expand into adjacent structures. In the alveolar neoplastic regions, cuboidal or columnar cells replace the normal type II pneumocytes, but the structure of the alveolar wall is maintained (lepidic growth) (Figure 2F). Concurrently, polypoid ingrowths arise from the bronchiolar epithelium in affected terminal bronchioles (Figure 2G). The stroma of the tumour is generally thin but may be infiltrated by variable amounts of lymphocytes, plasma cells and connective tissue fibres. Macrophages are consistently found in variable numbers surrounding neoplastic alveoli and affected bronchioles. Neutrophils can also be found, but they are interpreted as indicative of secondary bacterial infections. In some cases, mesenchymal tissue foci (myxoid nodules or growths) have been described admixed with the neoplastic epithelial component [25,27,45]. The histopathological features of atypical OPA are essentially the same as those of classical OPA, but a large number of inflammatory cells and connective fibres infiltrate the stroma. The histological appearance of experimentally induced OPA closely resembles that of natural cases [25].

### 3.3. Aetiology and Pathogenesis

Jaagsiekte sheep retrovirus is the causative agent of OPA [23,24]. This retrovirus infects and induces the transformation of secretory epithelial cells of the distal respiratory tract of sheep, and more rarely of goats and wild mouflon [25]. JSRV is an exogenous retrovirus belonging to the genus Betaretrovirus and is highly related to enzootic nasal tumour virus (ENTV) of sheep (ENTV-1) and goats (ENTV-2), which also causes an adenocarcinoma of secretory cells of respiratory epithelia, but in the upper tract [46,47]. Interestingly, sheep and goats, and other mammals, contain several copies of nonpathogenic JSRV-related endogenous retroviruses (enJSRVs) integrated in their genome [48,49]. JSRV has the typical genomic organization of a simple retrovirus, and contains the genes *gag*, *pro*, *pol* and *env*. These genes, respectively, encode the proteins of the viral core (MA, CA, NC and others), the viral protease (PR), the viral reverse transcriptase (RT) and integrase (IN) enzymes and the glycoproteins of the viral envelope (the surface domain SU which interacts with the cellular receptor and mediates cellular entry, and the transmembrane domain TM). In addition to encoding viral envelope proteins, the *env* gene of JSRV functions as a dominant oncogene. Its sole expression is sufficient to induce cellular transformation [50,51], and the cytoplasmic tail of the JSRV TM protein is essential for envelope-induced (Env-induced) transformation [52]. Apart from these four common retroviral genes, JSRV has a further open reading frame (orf-x) overlapping *pol* gene, whose role is unknown. Noncoding regions are present at the ends of the genome: U5 is present at the 5′ end, U3 at the 3′ end and the R region is repeated at both. Once JSRV interacts with a specific cellular receptor to enter the cell (HYAL2) [53], and after reverse transcription of the viral genome into double-stranded DNA, the viral DNA integrates into the host DNA to form a provirus. During the process of reverse transcription, the noncoding regions at the ends of the genome are duplicated and give origin to the viral long terminal repeats (LTRs), which are major determinants of retrovirus tropism. JSRV can infect many cell types; in fact, it establishes a disseminated infection of the lymphoid tissues of OPA affected sheep [33], but the exogenous JSRV LTRs are particularly active in type II pneumocytes and Club cells of the lung, the cells where the tumour develops [54].

The mechanisms involved in JSRV Env-induced transformation have not been fully elucidated, but several studies have shown the activation of signalling pathways that control cellular proliferation, including phosphatidylinositol 3-Kinase (PI3K)-Akt and mitogen-activated protein kinase (MAPK) [3,55], and additional pathways, including the AGR-2-YAPI-AREG axis, may also contribute to oncogenesis in this disease [55]. Other mechanisms, such as targeted integration of JSRV, cannot be totally excluded [3].

OPA has several features in common with lung adenocarcinoma of humans, including a similar histological appearance and activation of common cell signalling pathways, and additionally, the size and organization of human lungs are much closer to those of sheep lungs than to those of mice. This has led to the suggestion that OPA may be a valuable large animal model for the human disease [5,56,57,58]. This model can be informative for understanding cancer in humans and can identify and test the efficacy of new therapeutic interventions in a high reproducible system [58].

### 3.4. Diagnosis

As stated above, diagnosis of clinical OPA is possible by the detection of moist respiratory sounds and the presence of frothy lung fluid emitted from the nostrils. The overproduction of lung fluid is a characteristic clinical sign, and in the final stages of classical OPA, variable amounts of nasal discharge are obtained when the rear limbs are raised (“wheelbarrow” test) or the head is lowered. The diagnosis can be confirmed by the detection of JSRV RNA in lung fluid samples using reverse transcriptase PCR [33]. However, not all cases of OPA produce this fluid in detectable amounts, such as the early OPA, and also atypical OPA, even in advanced stages. In these cases, post-mortem examination is needed for OPA diagnosis and gross pathology and histopathological changes described above should be observed.

Lesions of OPA can be confirmed by immunohistochemical methods for the detection of JSRV proteins, using antibodies against proteins encoded by *gag* and *env* genes [59,60]. Immunolabelling is associated with the cytoplasm of transformed alveolar and bronchiolar cells (type II pneumocytes and Club cells, respectively) where JSRV replicates actively (Figure 2H). In addition, JSRV proteins have been demonstrated in myxoid nodules and also in the infiltrating lymphoreticular cells of some early OPA lesions [45].

JSRV proteins can be also detected in tumour homogenates by the Western blotting technique [61]. In addition, OPA tumours are always positive when tested by PCR techniques for the detection of JSRV genome [33]. These tests are based on the detection of JSRV RNA by reverse transcriptase PCR, but JSRV proviral DNA can also be specifically detected by PCR. In this case, primers are designed to amplify the U3 region of the JSRV genomic sequence, in which major differences with JSRV-related endogenous retroviruses that the sheep genome contains are located.

However, in vivo identification of OPA preclinical cases and lesion-free infected animals would be vital for the implementation of effective strategies for the control and eradication of the disease. Unlike other ovine retroviral infections, such as visna-maedi virus (VMV), the absence of a specific antibody response in JSRV infected animals [31,32] has precluded the use of diagnostic serological tests. The design of PCR techniques for the specific detection of JSRV provirus integrated in the sheep genome [33] revealed the presence of JSRV in lymphoid tissues and PBMC in clinically OPA affected sheep, in animals with preclinical OPA lesions and in lesion-free infected animals [34,35,36,37,38] and opened the door to the development of blood PCR tests for preclinical diagnosis of OPA. Although this PCR blood test is very specific, it has low sensitivity and provides an inconsistent detection of JSRV [35,37,38,62], probably due to the low proportion of infected cells in the blood [63]. Therefore, this test is not suitable to test individual animals for accreditation purposes but can be applied for the identification of infected flocks [37] and has been used in epidemiological studies [36,38]. Other tests have been investigated in order to improve the sensitivity in the detection of OPA preclinical cases. The same PCR test on bronchoalveolar lavage samples collected from live animals provides better results than the blood PCR test for the detection of early OPA (early visible lesions and microscopic OPA), and its sensitivity is 89% in comparison to the results of histological examinations [64]. However, this test does not detect lesion-free infected animals which may develop the disease in the future, and the practical difficulties in collecting the samples prevent its application at the field level. PCR testing of bone marrow aspirates collected in asymptomatic infected sheep has been attempted with negative results, although positively labelled cells were revealed by immunohistochemical methods in bone marrow samples collected at necropsy [65]. The same PCR when applied to colostrum and milk samples of JSRV blood positive lactating ewes with no signs of OPA disease did not seem to be more sensitive than the blood PCR test, and also provided an inconsistent JSRV detection throughout a lactation period [41]. Apart from PCR tests, transthoracic ultrasonography of both sides of the chest is another method that has been investigated for the in vivo detection of OPA lesions, in an attempt to eliminate this disease [66,67]. This test may be very helpful in reducing the OPA prevalence in a flock by identification and culling of affected animals. However, the test currently lacks sufficient sensitivity and specificity for the diagnosis of early stages of the disease. It is not able to detect lesions smaller than 2 cm and cannot clearly discriminate some nodular parasitic lesions or suppurative pneumonias that could be confused with OPA nodules. A negative scan cannot provide a guarantee that the animal is free of JSRV infection nor early OPA, and re-scanning is recommended in a short time, as tumours can develop much faster (a few months) than previously thought [68]. Therefore, eradication of OPA based only on this method seems to be unlikely. More recently, other approaches to detect preclinical OPA have been tested, such as reverse transcriptase PCR tests on nasal swabs. These seem to be more sensitive than the blood PCR test, but they have been proposed at flock level, not for testing individual animals [69]. Tests based on biomarkers are also in progress [69]. In this regard, in a recent study, levels of several tumour markers were found to be significantly higher in the blood of sheep with clinically suspected OPA and lesions confirmed by macroscopic and histopathological examination, than in lesion-free animals [70]. These tumour markers are thought to facilitate the diagnosis of OPA, but its possible usefulness in the diagnosis of subclinical OPA has not been investigated.

## 4. Enzootic Nasal Adenocarcinoma

Enzootic nasal adenocarcinoma (ENA, enzootic nasal tumour) is a contagious neoplasm of gland cells of the ethmoidal mucosa and is aetiologically associated with a betaretrovirus. This retrovirus is known as enzootic nasal tumour virus 1 (ENTV-1), and it is closely related but distinct to JSRV, which causes the ovine pulmonary adenocarcinoma, and sheep endogenous retroviruses [46]. ENA has been experimentally reproduced in sheep serving as a probe of the aetiological connection of ENTV-1 and this neoplasia [71].

### 4.1. Epidemiology

ENA has been recorded in all major areas where sheep are farmed, with the exception of Australia and New Zealand, and is apparently absent from the UK [72]. ENA has also been described in goats and is associated with another betaretrovirus similar to JSRV named enzootic nasal tumour virus of goats (ENTV-2) [47]. ENA in goats seems to be expanding in China, and ENTV-2 isolates showed significant genetic differences from reported European ones [73,74]. Pathological descriptions in these cases showed some particular aspects which may be related to the differences observed between the Chinese and European isolates [73,74]. In addition, a nasal adenocarcinoma in sheep associated with JSRV but not with ENTV-1 has been recently reported in Ireland [75].

Epidemiological data indicate that ENA occurs enzootically but with a very variable prevalence in affected flocks, ranging from 0.1–0.3% (low prevalence) to 2–15% (high prevalence). In affected flocks, the disease is found mostly in animals between 2 and 4 years of age. In our experience, the most common presentation of ENA in sheep is as single cases found from time to time in the same flock. However, in goats, frequently, a group of diseased animals is detected at the same time. No genetic, sex or breed predisposition has been reported [72].

### 4.2. Clinical Features and Pathology

**Figure 3:**
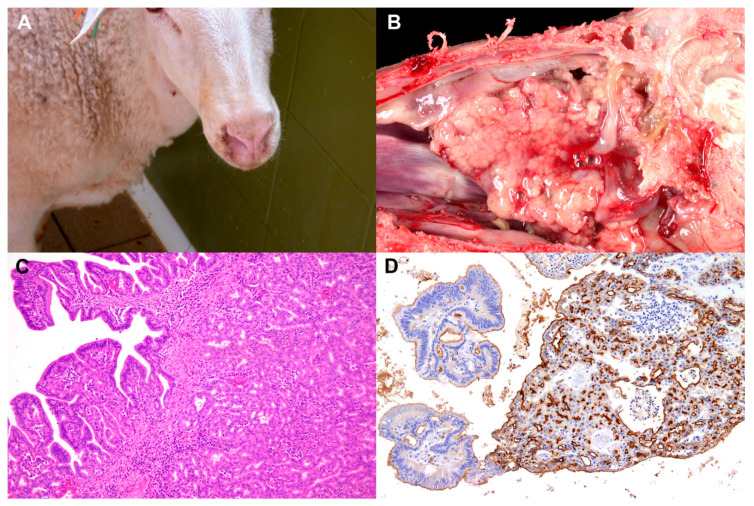
Enzootic nasal adenocarcinoma (ENA) clinical signs and pathology. (**A**) Sheep 3 years old. Right nasal nostril showing sero-mucous fluid discharge. As a consequence, right nostril looks cleaner and less hairy (washed nose). (**B**) Sheep 4 years old. Sagittal section of the skull showing replacement of normal ethmoidal turbinate by a multinodular or polypoid mass covered by mucus pressing close structures. (**C**) Histopathology of ENA. Epithelial cells proliferate into acinar and tubular patterns in inner parts; meanwhile, external zones show a predominant papillary pattern. Hematoxylin-Eosin. 100×. (**D**) Histological section of ENA. Immunohistochemistry. Monoclonal antibody against JSRV Env protein. Positive material is almost exclusively located on the apical membranes of both types of growths. 100×.

In naturally diseased sheep, the first clinical signs are not clear breathing problems, and a small amount of sero-mucous fluid emerging from the nostrils (unilaterally or bilaterally), dripping continuously from them, is the only detected change. As the disease progresses, more clear respiratory distress is observed with a variable amount of sero-mucous fluid stuck and bubbling around the nostrils when breathing. As respiration becomes more impaired by the tumour growth, snoring, coughing, sneezing, head shaking and mouth-breathing are seen. The increasing and continuous secretion of fluid causes a characteristic depilation on the area close to the upper lips (washed nose) (Figure 3A). As the tumour originates in the ethmoidal area, it expands either cranially inside the nasal cavity or laterally pressing close anatomical structures. As a result of this, close cranial bones are shown to be soft or atrophic, and the overlaid skin may protrude and eventually fistulize. Together with these changes, exophthalmos, as a consequence of the pressure on the retroocular structures, can be seen. In some cases, the tumour penetrates into the frontal sinuses expanding the cranial areas affected, and when tumours are bilateral, the clinical picture is much worse. The animals remain active and with a good appetite, and fever is usually not observed until near the time of death. Body condition is gradually lost, and animals become emaciated and eventually die due to septicaemic or toxaemic complications. The duration of the disease from the appearance of clinical signs to death has been reported to vary from 3 weeks to 9 months [72].

ENA has been experimentally reproduced in young lambs by nebulization of a mix of cell-free tumour extract. One of the infected lambs developed clinical signs similar to those described in spontaneous disease. Computer tomography showed a bilateral soft density tissue mass with poorly defined margins and few pinpoint areas of mineralization. The mass was causing destruction of the bony cartilages and nasal turbinates, filling around 50% of nasal cavity and associated with the accumulation of fluids within air spaces and sinuses [71].

Naturally occurring ENA has the characteristic clinical pattern described but can be confused clinically with other diseases, such as chronic proliferative rhinitis (CPR), oestrosis or other chronic bacterial or fungal infections. CPR is associated with *Salmonella enterica* subsp. *Diarizonae*, and the main lesion may look like a tumour, but lesions are mostly located in ventral turbinates and almost never in ethmoidal ones as in the case of ENA [76].

When performing the necropsy of the sheep with naturally occurring ENA, a sagittal section of the skull following the projection of nasal septum will result in optimal inspection of the nasal chambers. When opened, a tumour mass arising from the ethmoidal mucosa and effacing the normal architecture of the ethmoidal conchae is observed (Figure 3B). Tumours are generally multilobular, granular or soft surface; grey or reddish-white in colour; and can be found unilaterally, bilaterally, expanded on close areas or penetrating into nasal or frontal sinuses. On occasion, nasal septum and cribriform plates are eroded, but this is not a common finding. Tumours are found together with nasal inflammatory polyps in some cases, or containing areas of necrosis or purulent inflammation [72,77].

The microscopic architecture of the tumours is similar in all natural cases examined by us and joins the characteristics of a low grade adenocarcinoma of nasal glands [72]. Light microscopy reveals epithelial secretory cells proliferating into acinar, tubular, papillary or even solid patterns. Inner parts of the tumours look more tubular or acinar; meanwhile, external zones show a clear papillary pattern (Figure 3C). Apart from some areas of cellular atypia or local invasion, no signs of malignancy are seen, and the mitotic index is low. In addition, metastases in regional lymph nodes or in other organs have not been recorded. The stroma is very scanty but infiltrated, either scattered or grouped, by lymphocyte/plasma cells. Electron microscopy and histochemical studies have revealed that neoplastic cells, in general, correspond to serous, mucous or mixed gland cells and seem to originate either in olfactory or respiratory mucosal glands [72]. Glycohistochemical characterization of histologically normal nasal mucosa and ENA supports the suggestion that the tubular growth originates from the olfactory mucosa, whereas the papillary growth originates from respiratory mucosa. It is also indicated that goblet cells may have a role in the histogenesis of the papillary portion of ENA in sheep [78].

In the case of experimentally induced ENA, two separate components are described. The first type is characterized by an adenosquamous neoplastic proliferation and the second type by a papillary neoplastic growth. Both have an outer layer of differentiated stratified squamous epithelium. In the first type, the epithelium is connected with underlying glandular epithelial cells in proliferation that make up most of this component. The neoplastic glandular cells have a low mitotic index and slight degree of anisocariosis. The second region has a papillary appearance, and under the epithelium abundant fibrous tissue is observed [71].

### 4.3. Aetiology and Pathogenesis

ENTV-1 is a type of betaretrovirus demonstrated in ENA natural sheep tumours [79], and genomic sequence studies comparing it with ENTV-2 and JSRV concluded that these retroviruses are very similar but distinct and from JSRV-related endogenous retroviruses [46,80]. ENTV-1 showed a small number of differences, and comparison studies of the nucleotide and amino acid sequences of viral genes revealed that ENTV-1 from North America was highly homologous to the European isolates [80]. Thus, it seems that ENTV-1 shows little genomic variation, and it shares common pathogenic mechanisms with JSRV and ENTV-2. In this regard, these three viruses interact with mammalian cells through the HYAL2 (hyaluronglucosaminase 2) receptor for virus attachment and entry and can infect different cell types. HYAL2 is a ubiquitous membrane surface protein that belongs to hyaluronidases. However, ENTV-1 active replication is mostly restricted to nasal cavity epithelia where viral long terminal repeats (LTRs) are active. This active replication of the virus is important, because their oncogenic properties are due to envelope proteins. Several in vitro experiments have indicated that JSRV and ENTV-1 share common mechanisms of cell transformation. JSRV and ENTVs *env* gene products clearly activate a number of proteins involved in signalling cascades controlling cell growth and fate, such as Protein Kinase B (Akt) or Mitogen activated kinase (MAPK) signalling pathways, inducing permanent activation of these tyrosine kinase cell signalling routes and disturbing the control of cell growth and survival, leading to a growth advantage of deregulated cells. In spite of this, other mechanisms leading to transformation, such as targeted integration of the virus genome, cannot be totally ruled out [3,72].

### 4.4. Diagnosis

We have learnt that ENTV-1 is very similar to related endogenous and exogenous betaretroviruses, and these similarities would represent a problem in trying to develop reagents to identify specifically each of them. However, ENTV-1 showed the largest region of sequence divergence in the LTR, particularly in U3 [46,79,80], and several specific PCRs to amplify part of the LTRs regions have been developed to detect ENTV-1 genome [47,80,81]. Proviral DNA U3 PCR has been used in the analysis of the tissue distribution of ENTV-1 in diseased animals, and the virus is rarely found out of the tumour and not in PBMC [47]. We may conclude from these results that ENTV-1 is mainly confined to the tumour, and the likelihood of detecting the virus in PBMC is very low. Thus, a PCR blood test would not be reliable as a preclinical test or for the detection of ENTV-1 infection.

Specific RT-PCRs (U5 and gag regions) from North American ENTV-1 isolates performed on nasal exudates have been proposed as a part of an ante-mortem diagnostic test for ENA surveillance and eradication programs. In this study, RT-PCR on nasal swabs was correlated with pathology [81]. Therefore, ENTV-1 can be diagnosed by PCR in animals showing clinical signs or with tumours at very early stages, but detection of only infected animals is not currently possible.

The evaluation of the serological immune response in sheep with ENA has been limited and controversial. Studies using Western blotting found reactivity to recombinant JSRV capsid protein (JSRV-CA), as a Glutathion S-transferase (GST) fusion protein, in sheep sera from diseased animals. However, this reaction could be abolished completely by absorption with the GST fusion partner but not with JSRV-CA. This suggested that the activity recognized against this recombinant JSRV-CA was not specific [31]. In addition, Western blotting techniques also failed to detect any antibody against viral antigens from concentrated ENA fluid (De las Heras, unpublished results). A recent study identified ENTV-1 reactivity by ELISA and Western blot in sheep serum from ENA affected and in contact sheep belonging to a flock with high incidence of ENA. Serum samples were tested for reactivity against recombinant ENTV-1 capsid and surface subunit (Env) proteins produced using a polyhistidine tag (His-tag) bacterial expression system and removing His-tag prior to use it as an antigen. Results showed that reactive antibodies against both capsid and Env proteins could be detected in the serum of sheep either with or without tumour evidence using ELISA and Western blot analysis. Both techniques detected immune reactive antibodies against Env protein from the flock with a history of ENA but not in the naive sheep serum samples. Furthermore, some samples from these sheep were also positive to a neutralization test of ENTV-1 Env-pseudotyped virions. These results suggest that these interactions were indeed specific and that sheep are able to develop antibodies against the ENTV-1. In spite of this, ELISA and virus neutralization tests showed low specificity and sensitivity, and they are unreliable to be used to diagnose ENTV-1 infection [81]. Similar results have been obtained in sheep experimentally infected with ENTV-1 [82]. In any case, this is a very relevant finding because it means that sheep may not be immune tolerant to exogenous ENTV-1 infection as previously thought. Studies on cell-mediated immunity to ENTV-1 have not yet been reported [72].

Although ENTV-1 and JSRV are distinct viruses, translation and alignment of open reading frames suggest a very high degree of homology between them [79]. The similarity between the coding regions of these retroviruses is in agreement with the evidence that antisera against Env proteins of JSRV cross-reacted with ENTV-1 [60]. Thus, monoclonal antibodies (Mab) against the surface domain of the JSRV envelope protein (Env) were generated. Two of them (Clones C9 and B3) gave intense immunolabelling of lung tumours in sheep infected with JSRV, and in mice exposed to an adenoviral vector encoding Env (replication-defective adeno-associated virus type 6). These monoclonal antibodies recognized tumours in all JSRV-infected sheep examined but not any cross-reacting antigens in lung samples with a variety of diseases that were not the result of JSRV infection [60]. In addition, these antibodies also labelled tumour cells in mouse lung tumours induced by the same adenoviral vector encoding ENTV-1 envelope protein and in natural cases of ENA in sheep [60] and goats (De las Heras, unpublished results). Together, these results indicate that antibodies are highly specific for Env proteins of ovine betaretroviruses (JSRV, ENTV-1 and ENTV-2) and may provide a useful diagnostic test for these retroviruses using immunohistochemical (IHC) methods in contagious respiratory tumours of sheep and goats.

These monoclonal antibodies for Env proteins have been used to study nasal smears for the detection of ENTV-1. Although ENTV-1 infected cells could be identified by IHC staining of nasal smears, the specificity and sensitivity were low [81]. In tumour sections, Env staining appears to be almost exclusively localized to the apical membrane as opposed to that in OPA sections, which also appears at high levels in the cytoplasm of tumour cells [60] (Figure 3D). More recently, another JSRV Env monoclonal via peptide synthesis was generated, and assays demonstrated that it was able to recognize OPA tumours [83]. This finding adds a new resource of reagents which may be used in these contagious respiratory tumours associated with betaretroviruses in small ruminants. The inconvenience of the use of IHC for ENA diagnosis is that all of these reagents are not commercially available.

Finally, we would like to indicate that using this Mab, cross reactions between OPA and ENA in sheep samples have to be taken into account in the IHC analysis and interpretation. Thus, we have to consider that the coexistence of JSRV infection and ENA in sheep has been reported, JSRV proviral DNA was detected in ENA tumour samples [84] and nasal adenocarcinoma associated with JSRV but not with ENTV-1 has been described in sheep in Ireland [75]. Therefore, the possibility of coinfection with JSRV and the identification of nasal tumours with morphological similarities to ENA but related with JSRV and not ENTV-1 in sheep shows the necessity of using PCR in combination with IHC to reach an accurate diagnosis of ENA.

## 5. Conclusions

Several neoplastic diseases deserve attention as causes of weight loss and emaciation in sheep. SIA affects the gastrointestinal area and has epidemiological and economic relevance in Australia and New Zealand, but is also present elsewhere in the world. However, the aetiology is not known, and more investigation efforts are needed to clarify which factors are clearly related to it. OPA is present in most sheep-rearing countries and causes significant economic losses. The disease is a contagious lung cancer caused by the oncogenic betaretrovirus JSRV, and diagnosis is based on the virus detection in the tumour lesion by PCR and immunohistochemical methods. There are no serological diagnostic tests due to the lack of a detectable immune response in JSRV infected animals, and although several tests have been attempted for the diagnosis in live animals, they are not sensitive or specific enough for the detection of early stage and lesion-free infected animals. Working on improving early diagnosis and the identification of infected animals is essential for the implementation of effective strategies for OPA eradication. ENA is prevalent in some areas, mainly in Europe and North America, and is also an infectious disease. The betaretrovirus related with ENA (ENTV-1) can be detected using PCR techniques or immunohistochemistry in live animals bearing tumours but cannot be detected at pre-neoplastic stages. Immune response is occasionally detected in diseased animals, but no serological diagnostic tests are available. More efforts should be made to investigate the early stages of infection and dissemination in order to find a diagnostic test which can be used in control and eradication plans.

## Data Availability

Not applicable.
